# Flexible Silver/Polyaniline Schottky Diodes on a Polyethylene Based Elastomer: A Sustainable Approach for Advanced Electronics

**DOI:** 10.1021/acsaelm.4c02318

**Published:** 2025-04-10

**Authors:** Raj K. Vinnakota, Shaimum Shahriar, Arun Ghosh, David Summerlin

**Affiliations:** Department of Chemistry and Physics, Troy University, Troy, Alabama 36082 United States

**Keywords:** Schottky junction, Flexible diode, Polymer electronics, Thermionic emission, and Recyclable elastomer substrate

## Abstract

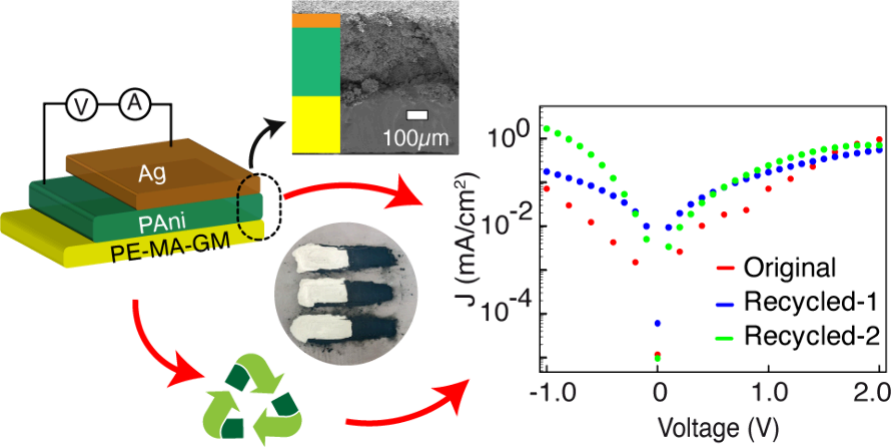

This study presents the fabrication and characterization of flexible Schottky diodes based on silver (Ag) and polyaniline (PAni) layers, constructed on a recyclable poly(ethylene-*co*-methyl acrylate-*co*-glycidyl methacrylate) terpolymer (PE-MA-GM) substrate. The fabrication process involved compression molding of the PE-MA-GM resin substrate and patterning of PAni layers using aluminum masks, followed by the deposition of Ag electrodes. Electrical characterization revealed stable current–voltage (I–V) characteristics at room temperature and under varying operating temperatures. Furthermore, the devices exhibited decent performance under mechanical bending at angles of 40°, and 60°, demonstrating robustness against mechanical stress. Additionally, we recycled entire devices and reconstructed Ag/PAni on the recycled substrate, achieving I–V characteristics comparable to the original measurements. While the fabrication methods used in this study were nonstandard, our preliminary findings underscore the potential of Ag/PAni Schottky diodes integrated onto a flexible and recyclable PE-MA-GM substrate. The results demonstrate promising performance metrics, with stable electrical behavior across varying operating conditions. This work offers an alternative approach to advancing the development of flexible and sustainable electronic systems. It paves the way for future studies focused on optimizing device performance, improving scalability, and expanding applications in wearable and environmentally conscious technologies.

## Introduction

Over the past two decades, polymer-based microelectronic devices have garnered significant attention for applications such as photovoltaic cells, organic light-emitting diodes (OLEDs), organic thin-film transistors (OTFTs), and field-effect transistors.^1−8^ Similarly, polymer-based electronics on flexible substrates have been extensively studied for use in wearable displays, Radio-Frequency IDentification (RFID) cards, detectors, and sensors.^9−11^ A critical component in these circuits is the Schottky diode, which forms a rectifying metal–semiconductor (M-S) junction and is essential for power electronics and energy harvesting.^12^ Understanding the electrical properties of the M-S junction is vital for optimizing the performance of polymer-based devices. Several studies have investigated the design and characterization of flexible Schottky barrier diodes (SBDs). A few studies in this area include organic flexible diodes with a Au/PEDOT:PSS (Poly(3,4-ethylenedioxythiophene):poly(styrenesulfonate))/Al structure,^13^ polymer-based diodes featuring a Pentacene–PEDOT:PSS interface, and Ag/ZnO diodes fabricated on polyimide substrates.^14^

Polyamide substrates are extensively used in flexible electronics due to their excellent mechanical flexibility, and thermal stability, making them ideal for robust and versatile applications.^15^ However, polyamide polymers are relatively rigid and less stretchable compared to elastomeric polymers. In flexible electronics, using recyclable elastomeric materials can provide several benefits of high flexibility and reversible stretchability of the devices. In contrast to traditional elastomers or rubbers, thermoplastic elastomers such as PE-MA-GM are thermomechanically recyclable and can be reformed to any structures. Notably, PE-MA-GM is chemically compatible with several ionic, polar, and nonpolar additives as needed during the fabrication of devices. PE-MA-GM combines the flexibility of ethylene and methyl acrylate with the reactivity of glycidyl methacrylate, offering toughness, flexibility, and chemical reactivity.^16−20^ The glycidyl methacrylate introduces epoxy groups, enhancing adhesion to various substrates and making it ideal for adhesives, polymer blends, and flexible electronics.^21^ Using recyclable materials like PE-MA-GM can significantly reduce the environmental impact and waste challenges associated with electronic devices.^22^ This highlights the need for alternative substrate materials that meet the performance demands of flexible electronics while addressing recyclability and sustainability issues posed by conventional polyamide-based substrates.

Building on the exploration of eco-friendly materials, conductive polymers, particularly PAni, have emerged as promising candidates for flexible electronics due to their low cost, processability, and inherent flexibility.^23^ PAni stands out among organic materials for its tunable electrical properties, ease of synthesis and processing, making it ideal for flexible electronic applications.^24−30^ Integrating PAni into Schottky diodes offers a pathway to develop flexible, recyclable, and efficient thermoelectric energy harvesters. Studies have shown that doping PAni with elements like tellurium enhances its thermoelectric performance, underscoring its potential in innovative energy harvesting solutions.^31−33^ Furthermore, PAni’s flexibility enables the fabrication of devices suitable for diverse applications, including smart textiles and portable electronics, broadening its role in emerging technologies.^32,33^

In this work, we present preliminary findings on the fabrication of Ag/PAni Schottky diodes on PE-MA-GM substrate. The device demonstrated decent electrical performance even under bending conditions, showcasing its robustness for flexible electronics. Additionally, temperature-dependent analysis revealed the potential of these diodes for thermal sensor applications. This aligns with the growing demand for Schottky devices with dual functionality, combining efficient I–V rectification with thermal energy detection. Such dual-capable devices simplify design complexity, reduce material usage, and enhance energy efficiency, making them particularly valuable for applications in wearable electronics, Internet of Things (IoT) devices, and energy-autonomous systems, where compactness and multifunctionality are crucial. These results highlight the promise of Ag/PAni Schottky diodes in advancing sustainable and versatile electronic technologies.

## Experimental Section

**Figure 1:**
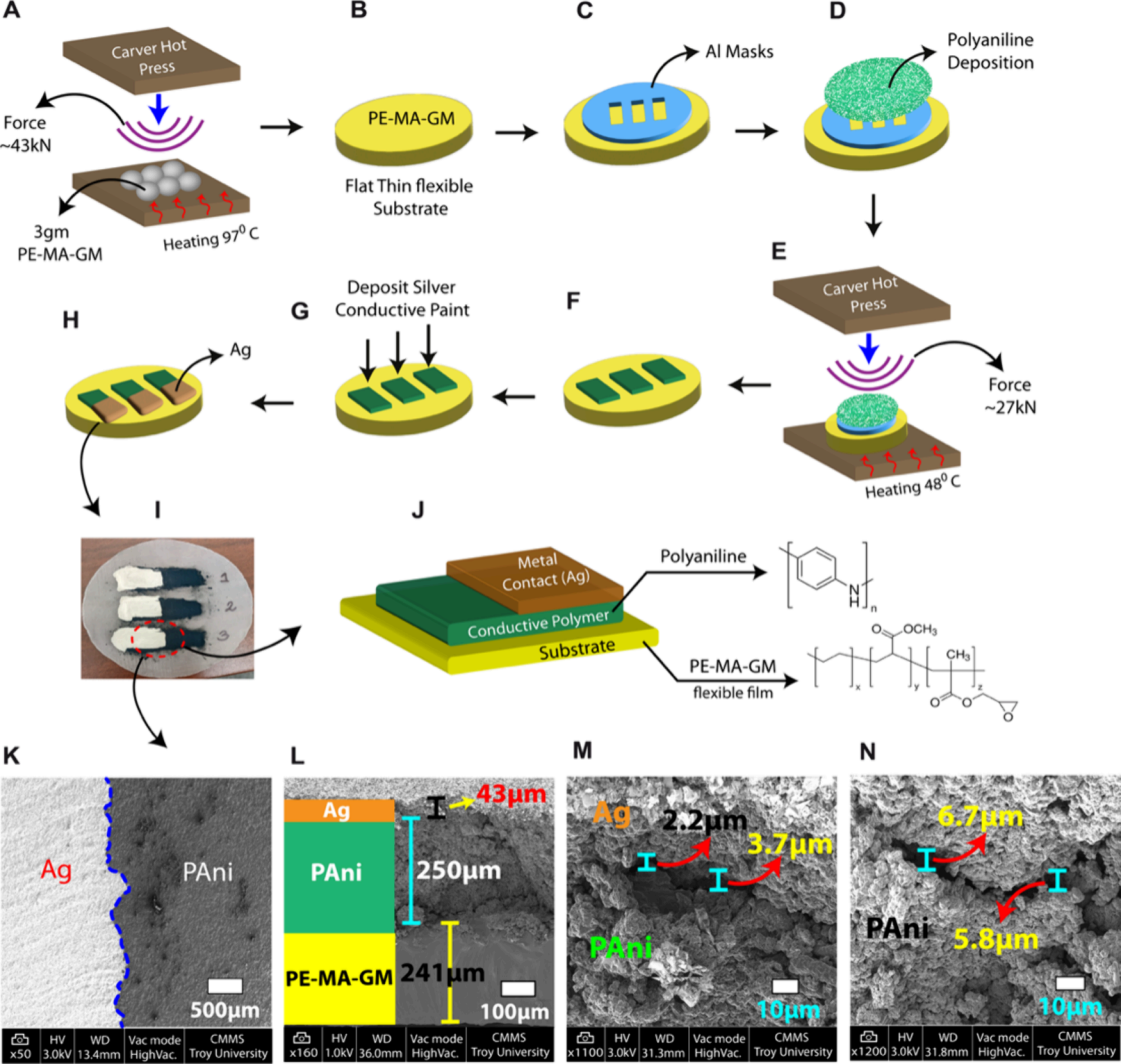
(A) Preparation of the PE-MA-GM substrate using a Carver Press. (B) Resulting flexible PE-MA-GM film. (C) Positioning of Al foil masks featuring three rectangular openings, each measuring 2.54 cm × 1.27 cm, onto the substrate. (D) PAni deposition onto the masked substrate. (E) Imprinting of PAni onto the PE-MA-GM film using a heated press. (F) Formation of PAni strips aligned with the masked regions. (G, H) Application of conductive Ag ink to create metal contacts. (I) Photograph of the fabricated sample with three Ag/PAni contacts. (J) Schematic illustration of the final device, highlighting the PAni and PE-MA-GM chemical structure. (K) Top-view SEM image of the device. (L) Cross-sectional SEM image displaying the layer thicknesses: Ag (43 μm), PAni (250 μm), and bottom flexible film (241 μm). (M) Highlighting the interfacial layer between Ag and PAni, with an average thickness of 2.95 μm. (N) Displaying some cracks in PAni layer, with an average thickness of 6.25 μm.

Poly(ethylene-*co*-methyl acrylate-*co*-glycidyl methacrylate) terpolymer (PE-MA-GM) with methyl acrylate content of 22–28% and glycidyl methacrylate content of 7–9 wt % was obtained from Sigma-Aldrich, Saint Louis, MO. Polyaniline or PAni (emeraldine salt) powder (infusible) with a particle size of 3–100 μm and an average molecular weight of >15,000 was also obtained from Sigma-Aldrich, St. Louis, MO. Aluminum foil was used for creating mask. Silver conductive paint (842AR) from MG Chemicals Ltd., Ontario, Canada, was used as the metal electrode in this study without any modifications. The paint consists of 50% silver flakes as the conductive filler, dispersed in a solvent-based acrylic lacquer binder. The remaining composition includes dimethyl carbonate (16%), acetone (11%), heptan-2-one (10%), and 1-methoxy-2-propanol acetate (1%), which serve as solvents to optimize viscosity, adhesion, and drying properties. The fabrication process flow for the Ag/PAni Schottky diodes is illustrated in Figure 1.

The devices were constructed on a flexible substrate made of PE-MA-GM. The process began with the preparation of the substrate material. Specifically, 3 g of PE-MA-GM resin were melted at 97 °C and compression molded at 43.14 kN for 3 min using a Carver Bench Top Standard Heated Press (Model 4122). This step produced a flat, thin, and flexible film, suitable for subsequent device fabrication. To pattern the PAni layer on the flexible substrate, three rectangular masks (2.54 cm × 1.27 cm) were created from aluminum foil. These masks facilitated the deposition of three isolated PAni strips on top of the substrate, enabling the simultaneous fabrication of multiple devices. The PAni layer was imprinted onto the PE-MA-GM film using a 1:3 mass ratio of PAni to substrate material. The same Carver press was used, with the temperature maintained at 48 °C and a force of 26.69 kN applied for 2 min. This process resulted in thin, uniform PAni strips corresponding to the masked areas, forming the basis for the planar Schottky diode structure. To complete the device fabrication and establish the metal–semiconductor junction, a conductive Ag layer was deposited onto the PAni strips. Conductive Ag paint was carefully applied to a selected portion of the PAni surface using a fine brush. As the solvent evaporated, the silver flakes appeared to form a continuous conductive network, potentially facilitating charge transport in the Schottky diode. The acrylic lacquer binder likely contributed to mechanical stability while promoting adhesion to the polyaniline substrate. The paint dried at room temperature within approximately 3 min, allowing for relatively rapid processing while maintaining conductivity. This formulation was selected due to its reported adhesion, conductivity, and compatibility with flexible electronics, which may support charge injection and mechanical integrity in the polyaniline-based Schottky diode. Although this method differs from conventional metal deposition techniques, it provided reasonable control over the process, potentially minimizing the risk of overspreading and ensuring a well-defined metal–semiconductor junction. The resulting junction area was measured at approximately 3.2 cm^2^. The final SEM images (see Figure 1(L-M)) provide a comprehensive structural analysis of the device. The top-view image illustrates the overall surface morphology, while the cross-sectional view details the layer thicknesses: Ag: 43 μm, PAni: 250 μm, and the flexible film: 241 μm. Additionally, the interface between Ag and PAni reveals microsized gaps and localized contact points, which can serve as potential charge transport pathways, with an average interfacial layer thickness of 2.95 μm. Moreover, we observed cracks at certain regions, some appearing more uneven than others. To highlight this, we zoomed in on a specific crack within the PAni layer, measuring an average thickness of 6.25 μm, providing further insight into the structural integrity and potential impact on device performance.

## Characterization

**Figure 2:**
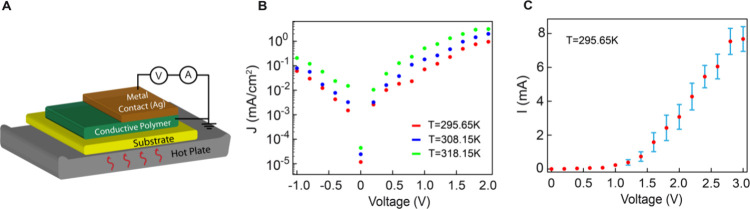
(A) Schematic of the experimental setup used to measure the I–V characteristics of the device under varying temperatures. For this setup we used Keithley 6517B Electrometer/High Resistance Meter for conducting I–V measurements, with the temperature controlled using a calibrated hot plate. (B) Semilog plot of the measured J versus V for the device at various operating temperatures. The figure shows the forward and reverse characteristics of the Ag/PAni contact, highlighting the temperature dependence of its electrical behavior. (C) The I–V plot depicts a standard deviation based on measurements from three samples each containing three Ag/PAni Schottky diodes, totaling nine measurements, ensuring statistical reliability. For simplicity, only the room temperature plot is included.

The electrical characterization of the fabricated Ag/PAni Schottky diodes was carried out to assess their performance under varying conditions. Figure 2(A) illustrates the schematic of the experimental setup used for measuring the I–V characteristics of the device at different temperatures. The Keithley 6517B Electrometer/High Resistance Meter was employed for voltage and current measurements, with the meter configured to perform a sweep of the I–V characteristics. Additionally, temperature-dependent I–V measurements were conducted by applying a controlled temperature gradient using a calibrated hot plate setup. The electrical characterization was conducted on a total of three samples, each containing three Ag/PAni Schottky diodes, ensuring decent statistical reliability. Figure 2(B) presents the semilog plot of the measured and averaged forward and reverse bias current density (J) versus voltage (V) for the device at various operating temperatures. The current density was calculated using the standard procedure of dividing the measured current by the contact area of the diode, which, in this case, is 3.2 cm^2^. To assess measurement consistency and variability, Figure 2(C) presents the standard deviation, offering insights into the reproducibility of the observed I–V characteristics. For simplicity, only the plot at room temperature is included. Here, we observe a larger standard deviation, primarily influenced by fabrication-related factors, including (i) uncontrolled variations in the polyaniline (PAni) layer thickness across different samples, (ii) nonuniform Ag application due to the brush deposition method, leading to contact and thickness inconsistencies, and (iii) inherent challenges in precisely controlling the thickness of the intermediate (Ag/PAni) insulation layer.

## Results and Discussion

To investigate the electrical behavior of the fabricated device and extract critical parameters such as the Schottky barrier height (ϕ_*B*_), ideality factor (*n*) and the effective Richardson constant (A*), current–voltage measurements were recorded and analyzed. It is well-known that the charge carrier transport across a Schottky junction is governed by majority carriers and can occur through three primary mechanisms: carrier diffusion, quantum-mechanical tunneling, and thermionic emission (TE) over the Schottky barrier. While these mechanisms can coexist, thermionic emission typically dominates the charge transport in most Schottky interfaces. TE process is described by eqs 1 and 2(34−49) when the forward bias voltage; *V* > 3*k*_*B*_*T*/*q*. According to TE, it is assumed that the current is primarily controlled by carrier transport across the metal–semiconductor interface, with the drift and diffusion of carriers within the depletion region playing a negligible role.
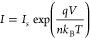
1
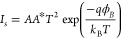
2Here, *I*_*s*_ represents the saturation current, *A* denotes the Ag/PAni contact area (measured 3.2 cm^2^ in this study), *q* is the elementary charge, *k*_*B*_ is the Boltzmann constant, and *T* is the operating temperature. When analyzing Schottky barrier diodes (SBDs), it is crucial to consider the fabrication process. This is because many SBDs inherently feature a thin interfacial layer, often a native oxide, between the metal and the semiconductor, unless deliberate measures are taken to prevent its formation. As depicted in the SEM image in Figure 1(M), our device features an interfacial layer approximately 2.95 μm thick. The presence of this insulating layer effectively alters the device structure, converting it into a Metal–Insulator-Semiconductor (MIS) configuration.^50−56,12,57^ This transformation can have a substantial impact on the diode’s electrical behavior, influencing critical performance parameters, such as the ϕ_*B*_, *n* and A*. Moreover, this thin insulating layer can alter carrier dynamics at the metal–semiconductor interface, potentially affecting the device’s overall performance. This impact arises from the interfacial layer introducing interface states.^50−56,12,57^ In our current work, the fabrication process employed deviates from standard methods commonly used in metal–semiconductor device fabrication, such as spin coating, doping techniques, thermal evaporation, or sputtering techniques. Instead, we utilized a nonconventional approach that prioritizes simplicity and exploratory analysis. Given this unconventional method, the potential impact of such a thin interfacial layer, on the device’s electrical parameters is expected to be significant. This underscores the importance of carefully accounting for the presence and characteristics of such layers when interpreting the electrical behavior of the fabricated device.

Considering the fabricated device as an MIS structure, the total externally applied potential (*V*) is distributed across multiple regions of the device, including a voltage drop (*V*_*s*_) across the interfacial layer, often modeled as a series resistance (*R*_*s*_). The presence of this *R*_*s*_ is critical, as it introduces a nonideal behavior in the device’s electrical characteristics.^46,54^ However, this deviation from ideality is particularly pronounced at higher forward-bias voltages, where the voltage drops across the series resistance becomes significant. The forward-bias IV characteristics of an SBD, incorporating the effects of series resistance (*R*_*s*_), are described by the modified thermionic emission model^46,49−51,54,58^ where the total current is expressed as
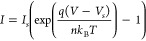
3
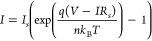
4

**Figure 3:**
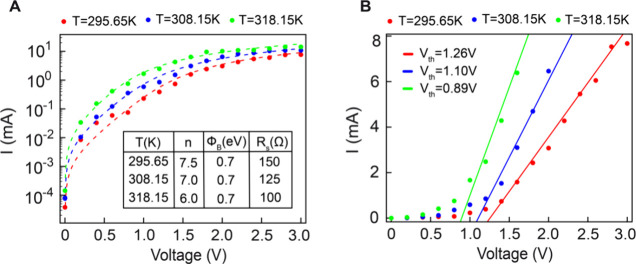
(A) Semilog plot (log(I) vs V) of the temperature-dependent forward-biased current–voltage (I–V) characteristics of the device at *T* = 295.65 K, 308.15 K ,318.15 K. Experimental data points are shown as dots, while the dashed curves represent the theoretical fitting results. The figure illustrates the influence of temperature on I–V behavior, with the corresponding fitting data provided in the inset. (B) I–V plot with linear fit used to extract the threshold (*V*_*th*_) or forward voltage for the device. The extracted threshold voltages are *V*_*th*_ = 1.26 V at *T* = 295.65 K, *V*_*th*_ = 1.10 V at *T* = 308.15 K and *V*_*th*_ = 0.89*V* at *T* = 318.15, illustrating the variation of the threshold (or forward) voltage with temperature.

Fitting of the measured forward biased IV data to theoretical IV curves was performed by using *R*_*s*_, *n*, ϕ_*B*_, and A*as fitting parameters. The metal contact area for the device is *A* = 3.2 cm^2^. The comparison between the experimental and fitted curves is presented in Figure 3(A). The ideality factor (*n*) is an important parameter for characterizing the charge transport mechanism. The fitting results (see inset in Figure 3(A)) demonstrate a strong correspondence between the experimental I–V data and the theoretical fitting curves, suggesting that the chosen fitting parameters are decent and appropriately applied. The extracted effective Richardson constant is A* = 5*A*/cm^2^ K^2^. The close alignment of the fitting curves indicates that the model effectively captures the device’s electrical behavior under the tested conditions. However, upon examining the extracted fitting parameters (as shown in inset Figure 3(A)), it is observed that the ideality factor is relatively high, with a value of 7.5 at room temperature (*T* = 295.65 K). This elevated ideality factor indicates deviations from the typical thermionic emission behavior, where the ideality factor (*n*) generally falls within the range of 1 to 2. However, the observed elevated ideality factor suggests the presence of additional charge transport mechanisms beyond thermionic emission. These mechanisms could include quantum tunneling or recombination in the depletion region (carrier recombination at defect sites within the depletion region).

As observed in the SEM image in Figure 1(M), the interfacial layer also features localized contact points between the Ag and PAni. These nonuniform contact regions likely lead to the formation of multiple thin Schottky junctions, where certain areas facilitate direct metal–semiconductor contact while others are separated by a varying interfacial layer thickness. Such variations can create localized tunneling pathways, enabling quantum mechanical tunneling of charge carriers through the barrier. This tunneling effect significantly contributes to the overall current transport mechanism, increasing the deviation from ideal thermionic emission behavior and leading to an abnormally high ideality factor. Additionally, the presence of interface states and trap-assisted recombination further exacerbates this deviation, reinforcing the nonideal characteristics of the diode. Defect states at the interface may act as recombination centers, facilitating carrier recombination within the depletion region and thereby further increasing the ideality factor. This combination of tunneling effects and recombination mechanisms highlights the complex nature of charge transport in the device beyond standard thermionic emission theory. Thus, the high ideality factor may be attributed to the nonstandard fabrication process used in this study, including the pressing technique and the application of metal contact via a paintbrush method. While this approach is suitable for initial proof-of-concept investigations, it may have introduced imperfections such as interfacial defects and inhomogeneities in the ϕ_*B*_. These imperfections could lead to carrier diffusion through intermediate layers, resulting in a significant potential drop and an increase in *R*_*s*_, as observed in the fitted data (see inset in Figure 3(A)). Additionally, the irregularities in barrier height across the contact area, caused by fabrication-induced inconsistencies, may create localized conduction pathways with nonideal behavior, further raising the ideality factor.

To validate the reliability of the fitting results, the recorded I–V data at two additional operating temperatures, 308.15 and 318.15 K, were also analyzed using eq 4, as illustrated in Figure 3(A). As summarized in Table 1, the extracted ϕ_*B*_ remains consistent at approximately 0.7 eV, a value that aligns closely with those reported in the literature for polymer-based Schottky diodes.^59−61^ The analysis further reveals a significant temperature dependence in both the ideality factor (*n*) and *R*_*s*_, with both parameters decreasing as the temperature increases. This trend underscores the thermally activated nature of charge transport mechanisms across Ag/PAni interfaces, as observed in previous studies for M-S junctions.^42,44,47^ At elevated operating temperatures, current flow across the Ag/PAni junction becomes increasingly dominated by localized interfacial regions. This behavior is likely influenced by the nonstandard fabrication process employed, which may have introduced nano- or microscale interfacial patches (See SEM image in Figure 1(M)). These regions contribute to inhomogeneities in the Schottky barrier height and result in elevated local ideality factors. Such irregularities facilitate the formation of localized conduction pathways, leading to nonuniform charge transport across the Ag/PAni interface and deviations from ideal diode behavior. As a result, the dominance of thermionic emission as the primary conduction mechanism is disrupted, causing deviations from the ideal diode behavior. This phenomenon aligns with findings from extensive studies, which underscore the significant impact of barrier height inhomogeneities on the electrical performance of metal–semiconductor junctions.^48,49^

As the operating temperature *T* increases, the intrinsic carrier concentration (*n*_*i*_) in the PAni region rises significantly. This increase is attributed to the temperature-dependent exponential term in the *n*_*i*_ equation.^12^ Consequently, the saturation current (*I*_*s*_) also increases. The increase in *I*_*s*_ compensates for the reduction in the exponential term in eq 4, leading to higher diode currents at the same applied external voltage. This temperature-dependent behavior results in a noticeable leftward shift of the I–V curve, as illustrated in Figure 3(B). To evaluate the diode’s performance further, the linear region of the forward-bias I–V curve was analyzed to extract the threshold voltage (*V*_*th*_), representing the minimum voltage required to facilitate significant current flow across the Schottky junction. The linear fits, depicted as solid lines in Figure 3(B), demonstrate excellent correspondence with the experimental data. The threshold voltage decreases with rising temperature, with extracted values as follows: *V*_*th*_ at 295.65 K = 1.26 V, *V*_*th*_ at 308.15 K = 1.10*V* and *V*_*th*_ at 318.15 = 0.89 V. This temperature-dependent decline in threshold voltage is consistent with the typical behavior observed in Schottky diodes.^62^ Furthermore, as we observe from Figure 3 (B), maintaining a constant diode current reveals a decrease in the corresponding diode voltage with increasing temperature. For silicon-based diodes, this voltage change is typically around, – 2 mV/°C. In this study, the observed rate of change is approximately −12 mV/°C. This observation confirms the thermally activated behavior of the fabricated device, consistent with the expected temperature dependence of its electrical properties.

To further investigate the effect of the chemical composition of the Ag paint on the I–V characteristics, we considered the role of its key components. The acrylic lacquer primarily serves as a structural binder, while the conductive pathways are established by silver flakes. Once fully dried, the binder is not expected to significantly influence the electrical behavior of the device. To verify this assumption, multiple devices were tested over a 24-h period, and no noticeable variations in I–V characteristics were observed. This consistency suggests that the solvents and binder do not introduce electrical instability or interfere with charge transport. These findings confirm that the solvent-based acrylic lacquer does not degrade or alter the electrical performance of the Schottky diode contact after drying, ensuring stable and reliable conduction in the polyaniline-based device.

## I–V Characteristics of Intrinsic PAni vs Ag/PAni via Polaron–Bipolaron Model

**Figure 4:**
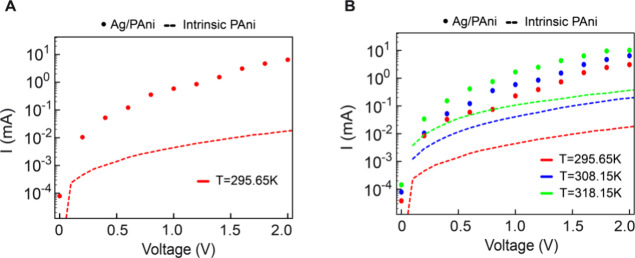
(A) Semilog plot (log(I) vs V) comparing the I–V characteristics of intrinsic PAni and the Ag/PAni Schottky diode. The intrinsic PAni curve highlights the polaronic and bipolaronic hopping transport, whereas the Ag/PAni Schottky diode exhibits rectifying behavior due to the formation of a Schottky barrier at the metal–semiconductor interface. (B) Semilog plot overlay of intrinsic PAni and Ag/PAni Schottky diode I–V characteristics at different temperatures, illustrating the temperature-dependent charge transport mechanism. The observed shifts in the I–V curves confirm the role of polarons and bipolarons in conduction, where increased temperature enhances carrier mobility, reduces threshold voltage, and improves current conduction.

To investigate the charge transport mechanism in the Ag/PAni Schottky diode, we first measured the I–V characteristics of intrinsic PAni without any metal contact. In its emeraldine salt form, PAni exhibits a nonlinear I–V response, primarily governed by the hopping transport of polarons and bipolarons. These charge carriers—polarons (singly charged radical cations) and bipolarons (doubly charged cations)—facilitate electrical conductivity through a thermally activated hopping mechanism,^63^ where transport is influenced by factors such as applied bias, operating temperature, and doping levels. As shown in Figure 4(A), the semilog plot (log(I) vs V) overlays the I–V characteristics of intrinsic PAni and the Ag/PAni Schottky diode, highlighting the nonlinear response of intrinsic PAni due to polaronic and bipolaronic hopping transport. This intrinsic PAni behavior serves as a reference for understanding charge transport in the Schottky diode, where the introduction of a metal contact significantly alters the transport dynamics. With the addition of the Ag contact, the I–V curve (red dotted line) exhibits strong rectifying behavior with an exponentially increasing current under forward bias, indicative of Schottky barrier-limited charge transport. This confirms the formation of a Schottky barrier at the Ag/PAni interface, where charge injection and transport are governed by barrier modulation and possible tunneling effects. The distinct shift in current response marks the transition from a hopping-dominated conduction mechanism in intrinsic PAni to barrier-controlled transport in the Schottky diode, demonstrating the critical role of the metal–semiconductor interface in modifying the overall electrical behavior. Thus, under the influence of forward bias, the applied voltage lowers the Schottky barrier, allowing polarons and bipolarons to move across the junction more efficiently. The presence of bipolarons facilitates a delocalized charge transport mechanism, effectively reducing resistance and enhancing current conduction. The comparison between intrinsic PAni and the Ag/PAni Schottky diode underscores the interplay between polaronic conduction and Schottky barrier modulation, reinforcing the notion that the nonideal I–V characteristics and the large ideality factor stem from a combination of charge carrier dynamics, interfacial effects, and tunneling-assisted transport.

Furthermore, Figure 4(B) also illustrates the temperature-dependent I–V response, providing additional confirmation of the role of polarons and bipolarons in the charge transport mechanism. At elevated temperatures, the increased thermal energy enhances the mobility of these charge carriers, leading to a reduction in the effective threshold voltage and an increase in current conduction.^64^ This is evident from the observed shifts in the I–V curves at higher temperatures, where thermally activated hopping and enhanced carrier injection facilitate improved transport across the junction. The comparison between intrinsic PAni and the Ag/PAni Schottky diode underscores the interplay between polaronic conduction and Schottky barrier modulation, reinforcing the notion that the nonideal I–V characteristics and the large ideality factor stem from a combination of charge carrier dynamics, interfacial effects, and tunneling-assisted transport. Higher temperatures contribute to the increased delocalization of bipolarons, further reducing resistance and enabling more efficient charge transport. The enhanced mobility of charge carriers lowers the effective Schottky barrier, allowing for a greater number of carriers to participate in conduction. Additionally, thermal energy assists in overcoming localized trap states at the metal–semiconductor interface, which can further contribute to the observed increase in current. This thermal activation effect explains the systematic shift in I–V characteristics with temperature variations, reinforcing the influence of polarons and bipolarons in defining the transport properties of the Ag/PAni Schottky diode.

## Effect of Mechanical Bending on Schottky Junction Characteristics

**Figure 5:**
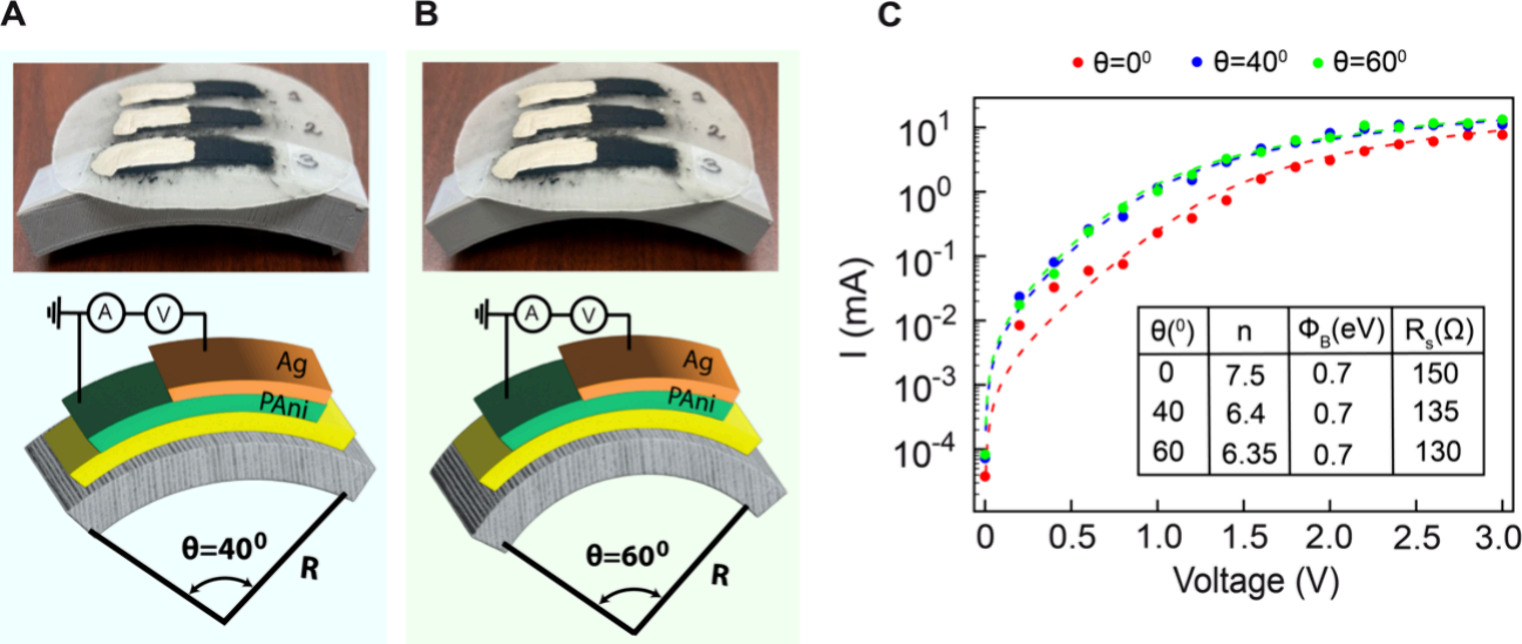
(A, B) Schematic representation of the bending experiment setup, alongside corresponding photographs showing the actual experimental setup for bending angles of 40° and 60°. The base of the bending apparatus was 3D printed using a MakerBot SketchLarge printer. (C) Semilog fitting plot for the forward-biased I–V characteristics of the device at different bending angles (θ = 40° and 60°). Experimental data points are depicted as dots, while the dashed curves correspond to the theoretical fitting results. The inset provides information about the fitting parameters and their correspondence to the experimental data. This figure highlights the dependence of the device’s I–V behavior on the stress induced by bending, demonstrating how the bending angle influences the electrical performance of the device.

To evaluate the performance of the fabricated device under mechanical strain, I–V characteristics were measured at bending angles of 40° and 60°. To achieve consistent bending conditions, custom-designed arced sample holders were modeled and fabricated using the Makerbot Sketch Large 3D printer. These holders were produced through Fused Deposition Modeling (FDM) with 1.75 mm polylactic acid (PLA) filament, selected for its excellent mechanical and thermal properties. These 3D-printed sample holders offer several advantages, including design flexibility, cost efficiency, and the ability to produce complex geometries tailored to specific experimental requirements. The use of 3D printing enabled quick prototyping and adjustments, ensuring the holders fit the desired bending angles. The samples were securely mounted on the 3D-printed holders (see Figure 5(A, B)), where the samples can be seen observed bent along a curve with a length of 11.3 cm, corresponding to surface curvature with radii *R* = 16.2 cm and 10.8 cm and bending angles of θ = 40° and 60°, respectively. Voltage and current measurements were performed using the Keithley 6517B Electrometer/High Resistance Meter, configured to execute a detailed sweep of the I–V characteristics.

**Figure 6:**
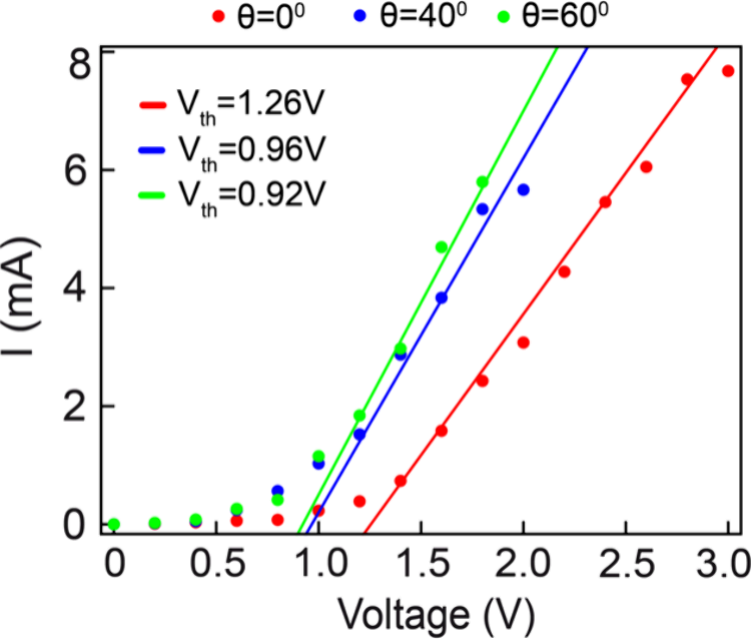
I–V plot with linear fit used to extract the threshold (*V*_*th*_) or forward voltage for the device. The extracted threshold voltages are *V*_*th*_ = 1.26 V at θ = 0°, *V*_*th*_ = 0.96 V at θ = 40° and *V*_*th*_ = 0.92 V at θ = 60°, illustrating the variation of the threshold (or forward) voltage with bending angles.

Figure 5(B) illustrates the semilog forward bias experimental I–V curves and corresponding fit to theoretical predictions using eq 4 to assess the impact of mechanical strain on the devices electrical performance. From the fitted parameters we observe that the critical parameters such as the ϕ_*B*_ and the ideality factor (*n*) exhibit a decrease with increasing bending angles. This behavior (decrease in ϕ_*B*_) can be attributed to the strain-induced modifications at the Ag/PAni interface, which significantly affect the Schottky junction’s properties. Tensile strain introduced during bending can lead to the formation of defects and trap states at the interface, resulting in localized inhomogeneities in the ϕ_*B*_. These inhomogeneities create regions with varying electrical properties, facilitating charge carrier transport and contributing to increased current flow under both forward and reverse bias conditions. Strain-induced changes significantly impact the potential distribution across the junction, leading to a reduction in the depletion region width and thereby altering the diode’s overall electrical behavior. These strain-induced effects are evident in the increased current observed at lower voltages, as shown in the I–V curves in Figure 5C. To further evaluate these effects, the linear region of the forward-bias I–V curve was analyzed to extract the threshold voltage (*V*_*th*_). The linear fits, depicted as solid lines in Figure 6, show a good agreement with the experimental data. The threshold voltage decreases with an increase in the bending angle, with extracted values as follows: *V*_*th*_ = 1.26 V at θ = 0°, *V*_*th*_ = 0.96 V at θ = 40° and *V*_*th*_ = 0.92 V at θ = 60°. This trend suggests that the observed reduction in threshold voltage is likely a direct result of the strain-induced phenomena described earlier, including changes to the junction’s potential distribution and the narrowing of the depletion region. These findings highlight the critical influence of mechanical strain on the device’s operational characteristics, emphasizing the need to understand strain effects for improving device performance in flexible and wearable electronics.^65^

The observed decrease in the ideality factor (*n*) with increasing bending angles indicates an enhancement in thermionic emission as the primary charge transport mechanism. This behavior aligns with the strain-induced reduction in the SBH, which lowers the energy barrier for charge carriers, thereby facilitating their transport across the junction via thermionic emission. Additionally, from fitting we observed that the mechanical strain introduced by bending also exerts an influence on the *R*_*s*_ of the device, as evidenced by a slight increase in *R*_*s*_ with greater strain. This increase in *R*_*s*_ can be attributed to changes in the mechanical integrity of the Ag/PAni interface under strain, potentially introducing contact irregularities or interfacial defects. The elevated *R*_*s*_ introduces additional voltage drops across the junction, further contributing to deviations from ideal Schottky diode behavior and impacting the I–V characteristics. The observed trends in *n*, ϕ_*B*_ and *R*_*s*_ are consistent with findings from other studies that investigated flexible Schottky diodes using different materials.^34,59^

## Fatigue Response and Charge Transport

**Figure 7:**
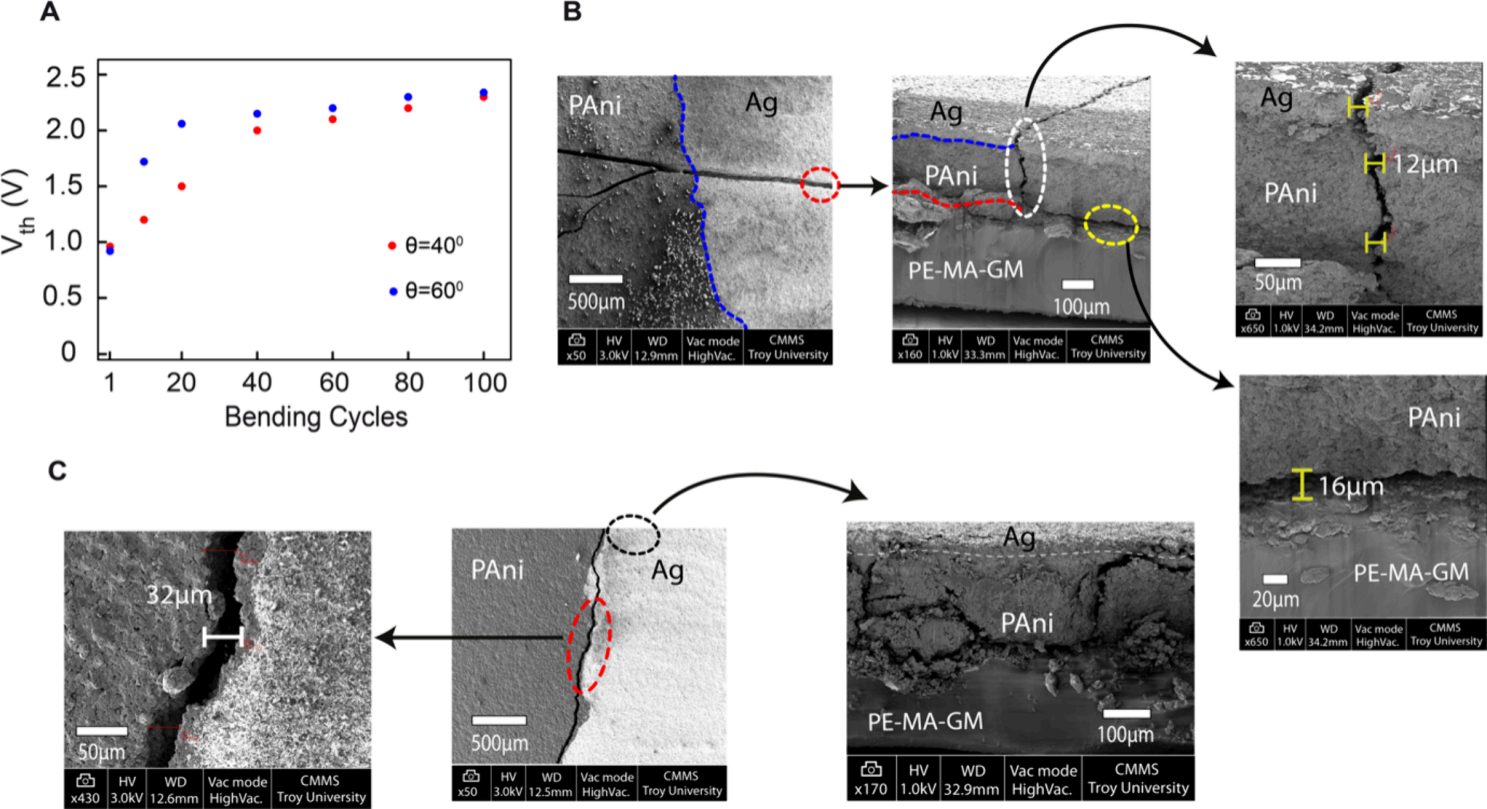
(A) Variation of threshold voltage for 40° and 60° bending angles as a function of bend cycles, highlighting the increase in threshold voltage due to mechanical deformation. (B) Top-view SEM image of the sample after 100 bending cycles at 40°, revealing crack formation perpendicular to the Ag/PAni interface. The cross-sectional SEM image further quantifies the crack dimensions, showing an average crack width of 12 μm in the Ag and PAni layers. Additionally, delamination of the PAni layer from the PE-MA-GM substrate is observed, with a measured peeling dimension of 16 μm. (C) SEM images of the sample after 100 bending cycles at 60°, showing a top-view SEM highlighting crack formation at the Ag/PAni interface. The cross-sectional SEM image confirms a separation crack width of 32 μm, along with visible peeling of the Ag layer from the PAni and the PAni layer from the base elastomer.

To assess the basic fatigue response of the Ag/PAni Schottky diode, we conducted a manual fatigue test, measuring I–V characteristics after controlled bending at 40° and 60° using 3D-printed fixtures. Due to the manual process, precise control over fatigue parameters such as frequency, strain variations, and force was not achievable. The test involved lifting and repositioning the sample to simulate repeated bending cycles. I–V measurements were taken after 1, 20, 40, 60, 80, and 100 cycles, with *V*_*th*_ extracted from linear fits. As observed in Figure 7A for both 40° and 60° bends, *V*_*th*_ increased, correlating with crack formation at the Ag/PAni interface, as seen in SEM images shown in Figure 7(B, C). The larger increase in *V*_*th*_ for 60° suggests greater bending-induced strain. This phenomena can be explained through polaron and bipolaron transport in PAni, where charge carriers rely on thermally activated hopping. Cracks disrupt these pathways, increasing resistance and the activation energy required for conduction, effectively raising *V*_*th*_. Separation at the Ag/PAni interface may also create localized depletion regions, further hindering charge injection.

SEM images after 100 cycles, as shown in Figure 7(B, C) reveal structural degradation. For 40° bending, top-view SEM shows a crack, while cross-sectional imaging confirms an average crack size of 12 μm in Ag and PAni, along with PAni delamination from the PE-MA-GM substrate (∼16 μm). For 60° bending, cracks at the Ag/PAni interface indicate layer separation, with a measured crack width of ∼32 μm. The increased damage aligns with the higher *V*_*th*_ observed. While surface SEM imaging shows cracks, subsurface conductive pathways may persist through residual adhesion, nanoparticle percolation, or tunneling effects. Since SEM cannot probe beneath the surface, further studies using cross-sectional TEM or conductive AFM are needed to fully understand postdeformation charge transport.

These results confirm that the Ag/PAni Schottky diode retains electrical functionality despite mechanical deformation. However, this is a qualitative fatigue assessment, and future work will include instrumented fatigue studies to evaluate long-term reliability. Efforts will also focus on improving Ag/Pani and PAni/PE-MA-GM substrate adhesion and refining fabrication for enhanced mechanical stability.

## Recycling, Reusability of the Device, and Future Efforts

Unlike traditional elastomers, PE-MA-GM exhibits thermoplastic properties, making it amenable to recycling through thermomechanical processes.^66^ The PAni emeraldine base, soluble in common organic solvents such as dimethyl sulfoxide (DMSO), dimethylformamide (DMF), and *N*-methyl-2-pyrrolidone (NMP),^67^ can be effectively removed, enabling the PE-MA-GM substrate to undergo recycling similar to other thermoplastics, such as polyolefins.^68−70^ PAni/PE-MA-GM systems can be corecycled to produce composite materials with enhanced thermomechanical properties, expanding their potential for applications. The rigid molecular structure of PAni can improve the mechanical stiffness and thermal stability of the PE-MA-GM elastomer, further enhancing the viability of reusing polymeric waste in high-performance applications.

The dual recyclability of PAni and PE-MA-GM underscores the environmental benefits of this system, supporting sustainable practices in electronic device development. The samples used in the above sections were recycled using traditional thermomechanical recycling methods. In the first approach, the PE-MA-GM flexible substrate was separated mechanically by removing the PAni and the Ag and extruded at 100 °C with a rotor speed of 100 rpm using a twin-screw extruder (Microcompounder MC 15 HT, Xplore, Sittard, The Netherlands). This recycled sample is designated as rPE-MA-GM. In the second approach, the entire device (Ag/PAni/PE-MA-GM) was cut into small pieces and blended with virgin PE-MA-GM (vPE-MA-GM) using a high-shear compounding method, where the material mixture had a 6:1 mass ratio of PE-MA-GM to Ag/PAni. The blending was conducted at 100 °C and 100 rpm for 10 min. Similarly, virgin PE-MA-GM was extruded through the same extruder. All samples were then compression molded at 100 °C for 5 min under a force of 43.14kN using a Carver press.

**Figure 8:**
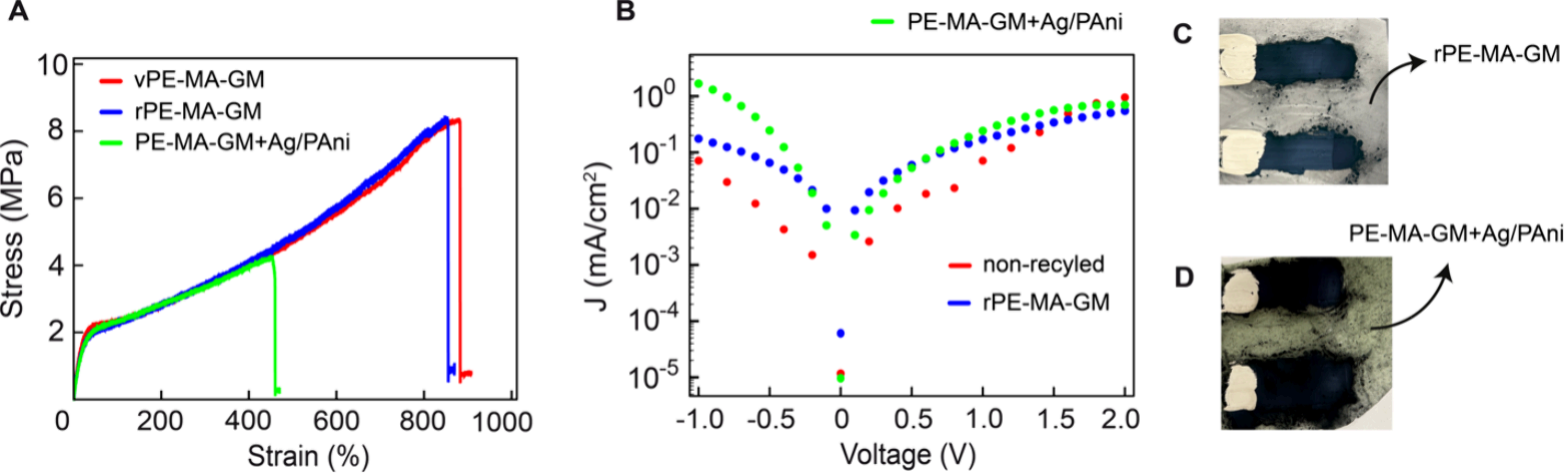
(A) Tensile stress–strain curves of different PE-MA-GM substrates: virgin PE-MA-GM (vPE-MA-GM) shown in red, mechanically separated and recycled PE-MA-GM (rPE-MA-GM) (blue), and fully recycled PE-MA-GM containing residual Ag/PAni components (green). (B) I–V characteristics of Ag/PAni devices fabricated on rPE-MA-GM (blue) and PE-MA-GM+Ag/PAni (green) compared to the original nonrecycled device (red). (C) Photograph of the Ag/PAni device fabricated on rPE-MA-GM. (D) Photograph of the Ag/PAni device fabricated on PE-MA-GM+Ag/PAni, showing noticeable discoloration to green, possibly due to residual Ag and PAni impurities retained during the recycling process.

The tensile stress–strain behavior of the materials was evaluated using a universal mechanical testing machine (Instron 5943, Norwood, MA) in accordance with ASTM D-638 standards. The machine was equipped with a 10 kN load cell, offering an accuracy of ± 0.5%. For the measurements, a gauge length of 15 mm and an extension speed of 10 mm/min were used. Each test specimen had an average width of 4.00 mm and a thickness of 0.20 mm. A minimum of five repeat measurements were conducted for each sample. The mechanical performance of rPE-MA-GM, PE-MA-GM+Ag/PAni, and vPE-MA-GM, particularly in terms of their tensile stress–strain properties, is critical for their application in flexible electronic devices. Several factors impact the mechanical performance of these materials, including their chemical structure, polymer–filler compatibility, filler size and surface reactivity, and the processing methods used. In this study, both virgin and recycled forms of PE-MA-GM exhibited similar tensile stress–strain behavior. However, the PE-MA-GM+Ag/PAni film demonstrated lower tensile stress–strain performance compared to the other samples. This reduction in mechanical performance is likely due to the presence of residual Ag and PAni impurities, which may have affected the material’s structural integrity. The average tensile strength ranged from 7.7 to 8.1 MPa, with an ultimate strain of 826% to 831%, and a Young’s modulus of 5 to 7 MPa (see Figure 8(A)). The mechanical performance of vPE-MA-GM is comparable to that of silicone elastomers, which are widely used in flexible electronic devices.^71−73^ However, unlike vPE-MA-GM, which is a recyclable thermoplastic, silicone elastomers are thermoset polymers and cannot be recycled in the same way as traditional thermoplastics.

To evaluate the recyclability of our device, we fabricated the base layer from the two different recycled substrates (denoted as rPE-MA-GM and PE-MA-GM+Ag/PAni). We then reconstructed Ag/PAni devices on these substrates (see Figure 8(C, D)) and performed I–V measurements to assess their electrical performance. Each I–V measurement was conducted on two separate samples per substrate, and the averaged I–V plots for the two recycled base substrates are presented in Figure 8(B). Despite the base layer undergoing the recycling process, the devices continued to exhibit decent I–V characteristics, demonstrating the feasibility of reusing the base material.

Additionally, the PE-MA-GM+Ag/PAni base substrate exhibited a noticeable color change after recycling, appearing green (as shown in the Figure 8D). This discoloration is likely due to residual PAni and Ag from the original device. To ensure that these residual materials did not introduce unwanted conductive properties to the base elastomer, we conducted separate I–V measurements on the recycled substrates alone. The results showed negligible current flow, confirming that the base elastomer remains nonconductive after the recycling process. This study underscores the recyclability and reusability of the device. The base elastomer can be effectively reclaimed and repurposed, offering a sustainable approach to device fabrication while minimizing material waste. Furthermore, to understand the adhesiveness between PAni/PE-MA-GM interfaces, an adhesion peel strength measurement was performed.^74^ The results showed an average adhesion strength of 350 N/m with a standard deviation of 34 N/m for the PAni/PE-MA-GM interface. It was measured according to ASTM Standard D903–2004 using an Instron universal testing machine, with an extension rate of 50 mm/min. Each sample was tested using five prepared specimens, and the averaged results along with the standard deviation are reported.

For future work, we aim to refine fabrication processes for improved uniformity and reproducibility, utilizing standardized techniques like spin coating for controlled film thickness and thermal deposition for reliable metal contacts. We will conduct comprehensive fatigue studies using specialized instruments such as Instron 5948 Micro-Mechanical Test System, which allow precise control of bending angles, speeds, and forces to assess mechanical durability under cyclic loading, with varying strain ranges and cycle counts. Additionally, efforts will focus on reducing the ideality factor (n) to enhance Schottky barrier properties and investigating the C–V response through polaronic charge transport modeling. We also plan to explore eco-friendly, recyclable substrates such as thermoplastic elastomers and biodegradable lignin-based materials to advance sustainable, flexible electronics. These efforts will optimize Ag/PAni Schottky diodes for applications in wearable sensors, energy-harvesting systems, and stretchable electronics.

## Conclusion

In conclusion, this study successfully demonstrates the design, fabrication, and characterization of a flexible Schottky diode using polyaniline (PAni) as the semiconductor material, PE-MA-GM as an elastomeric and recyclable substrate, and silver as the metal electrode. The device exhibits promising electrical characteristics, including distinct rectifying behavior with an exponential rise in current under forward bias and negligible leakage under reverse bias. Temperature-dependent analysis reveals a decrease in the threshold voltage with increasing temperature, indicating enhanced carrier injection and consistent performance across varying thermal conditions. Additionally, the device maintains its functionality under mechanical bending, without significant degradation in electrical performance. Furthermore, the reconstructed Ag/PAni on the recyled substrate, achieving IV characteristics comparable to the original measurements. The recyclable nature of the PE-MA-GM substrate aligns with sustainable practices, addressing the critical need for eco-friendly solutions in the electronics industry. These results position PAni-based flexible Schottky diodes as a promising candidate for integration into next-generation recyclable electronic systems, particularly in sensors that demand mechanical adaptability and environmental consciousness.

## Data Availability

All data supporting the findings of this article are available from the corresponding author upon reasonable request. All codes utilized in this article are available from the corresponding author upon request.
